# Characterization of Plant-Growth-Promoting Rhizobacteria for Tea Plant (*Camellia sinensis*) Development and Soil Nutrient Enrichment

**DOI:** 10.3390/plants13182659

**Published:** 2024-09-23

**Authors:** Mengjiao Wang, Haiyan Sun, Huiping Dai, Zhimin Xu

**Affiliations:** 1 School of Biological Science and Engineering, Shaanxi University of Technology, Hanzhong 723000, China; daihp72@snut.edu.cn; 2 Collaborative Innovation Center for Comprehensive Development of Biological Resources in Qinling-Ba Mountains, Hanzhong 723000, China; diyson2008@163.com; 3 Sanqin Talents, Shaanxi Provincial First-Class Team, Contaminated Soil Remediation and Resource Utilization Innovation Team at Shaanxi University of Technology, Hanzhong 723000, China; 4 School of Nutrition and Food Sciences, Louisiana State University, Baton Rouge, LA 70809, USA; zxu@agcenter.lsu.edu

**Keywords:** plant-growth-promoting bacteria (PGPR), rhizosphere soil, soil nutrients, plant growth, tea

## Abstract

Plant-growth-promoting rhizobacteria (PGPR) play an important role in plant growth and rhizosphere soil. In order to evaluate the effects of PGPR strains on tea plant growth and the rhizosphere soil microenvironment, 38 PGPR strains belonging to the phyla Proteobacteria with different growth-promoting properties were isolated from the rhizosphere soil of tea plants. Among them, two PGPR strains with the best growth-promoting properties were then selected for the root irrigation. The PGPR treatment groups had a higher Chlorophyll (Chl) concentration in the eighth leaf of tea plants and significantly promoted the plant height and major soil elements. There were significant differences in microbial diversity and metabolite profiles in the rhizosphere between different experimental groups. PGPR improved the diversity of beneficial rhizosphere microorganisms and enhanced the root metabolites through the interaction between PGPR and tea plants. The results of this research are helpful for understanding the relationship between PGPR strains, tea plant growing, and rhizosphere soil microenvironment improvement. Moreover, they could be used as guidance to develop environmentally friendly biofertilizers with the selected PGPR instead of chemical fertilizers for tea plants.

## 1. Introduction

Tea is one of the most popular beverages in the world and possesses many health benefits such as antioxidant, antibacterial, antimutagenic, and anticancer functions. Similar to other plants, the components of the rhizosphere soil microenvironment of tea plants, such as nutritional elements and rhizospheric microorganisms, can promote the remarkable growth of tea plants [1,2,3]. In recent years, many reports have pointed out that PGPR, as important rhizosphere functional microorganisms, can enhance plant growth and maintain plant health in many ways, such as dissolving insoluble phosphorus and potassium in soil, phytohormone production, enhancing water and nutrient uptake, reducing soil heavy metal toxicity, fixing nitrogen to improve nitrogen availability in the soil, producing ACC deaminase for ethylene breakdown, siderophore production, participating in the foundation of the anti-disease ability of plants, etc. [4,5,6,7]. Our previous study found that the rhizosphere soil microenvironment and plant growth could be improved by PGPR strain treatment [8].

In order to further understand PGPR strains and their effects on the growth environment of tea plants, the PGPR in the tea plant rhizosphere were isolated and identified. The growth-promoting capabilities of these PGPR strains were evaluated. Two PGPR strains with the best capabilities were chosen for the root irrigation to further confirm their tea plant-growth-promoting capability. The plant height and leaf chlorophyll content of tea plants, and the soil element content, microbial diversity in the rhizosphere, and metabolomics of tea plant roots were evaluated after root irrigation experiments. Correlations between PGPR strains with differentially accumulated metabolites (DAMs) of tea plant roots, rhizosphere microenvironmental factors, and physiological indices of tea plants were evaluated. The results of this study are helpful for us to further understand the mechanisms of PGPR affecting plant growth and the improvement of the rhizosphere soil microenvironment.

## 2. Results

### 2.1. The PGPR Strains in Rhizosphere Soil of Tea Plant

A total of 38 PGPR strains, which had a soluble phosphorus circle on inorganic phosphorus bacteria agar medium, were isolated from the collected soil samples. They were of the phyla Proteobacteria and classified into *Acinetobacter*, *Erwinia*, *Kluyvera*, *Leclercia*, *Pantoea*, *Pseudomonas*, *Rouxiella*, and *Serratia* (Table 1). The representative sequence of each group of PGPR strains is listed in Table 1.

**Figure 1:**
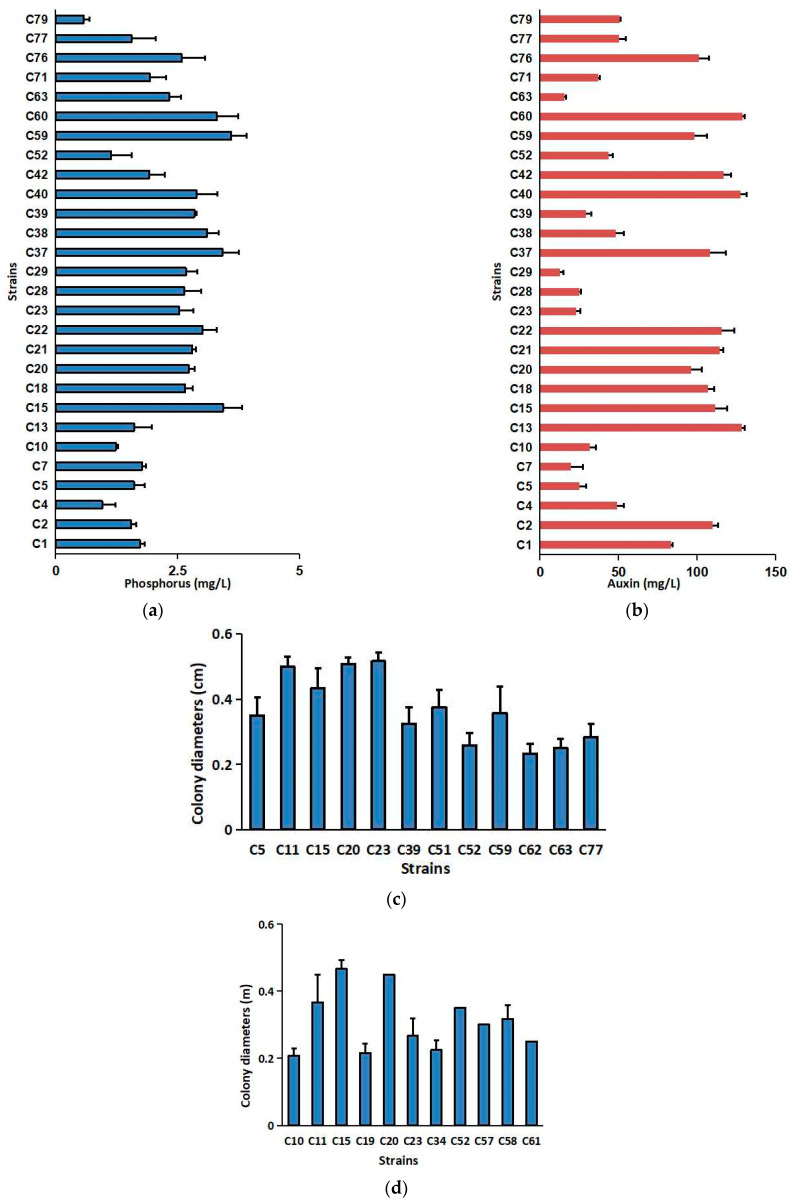
Growth-promoting characteristics of PGPR strains: (**a**) phosphorus-solubilizing capacity, (**b**) auxin production, (**c**) colony diameters of PGPR strains in silicate media, and (**d**) colony diameters of PGPR strains in A Sugai’s medium.

The phosphorus concentrations in the supernatants collected from the 28 cultured PGPR strains were higher than 0.50 mg/L, while the auxin concentrations of these 28 PGPR strains were higher than 12.00 mg/L (Figure 1a,b). Among them, the supernatant of strain C59 (*Erwinia* sp.) had the highest phosphorus concentration (3.59 mg/L) (Figure 1a). The highest auxin concentration (128.43 mg/L) in the supernatant was produced by strain C13 (*Erwinia* sp.) (Figure 1b). The typical phosphorus decomposition halos for PGPR strains are shown in Figure S1. The visible colonies were observed in the silicate bacteria media inoculated with strains C5, C11, C15, C20, C23, C39, C51, C52, C59, C62, C63, and C77, belonging to *Erwinia* sp., *Pseudomonas* sp., *Pantoea* sp., and *Serratia* sp. The C23 strain had the largest colonial diameter (5.17 cm) on the medium (Figure 1c). The colonies were also observed on A Sugai’s medium inoculated with strains C10, C11, C15, C19, C20, C23, C34, C52, C57, C58, and C61, belonging to *Erwinia* sp., *Pseudomonas* sp., *Pantoea* sp., *Rouxiella* sp., and *Serratia* sp. The C15 (*Erwinia* sp.) had the largest colonial diameters (4.67 mm) among all the strains (Figure 1d).

### 2.2. Effects of the PGPR Strains Used in Root Irrigation on Plant Growth and Soil Element Contents

Because of the high levels of phosphorus concentration (3.43 and 2.72 mg/L in the supernatant, respectively), silicate solubilization (0.43 and 0.58 cm colonial diameters in silicate bacteria medium, respectively), auxin production (111.59 and 96.27 mg/L in the supernatant, respectively), and nitrogen fixation (0.47 and 0.45 cm colony diameters in A Sugai’s media, respectively), strains C15 and C20 belonging to the *Erwinia* and *Serratia* genus were chosen for the root irrigation experiment.

The Chlorophyll (Chl) concentration and plant height of tea plants are shown in Figure S2. There was no significant difference in the Chl concentrations in the eighth leaves of tea plants between treatment and control groups (Figure S2a), while strains C15 and C20 significantly increased the plant height of treated tea plants (Figure S2b). 

**Figure 2:**
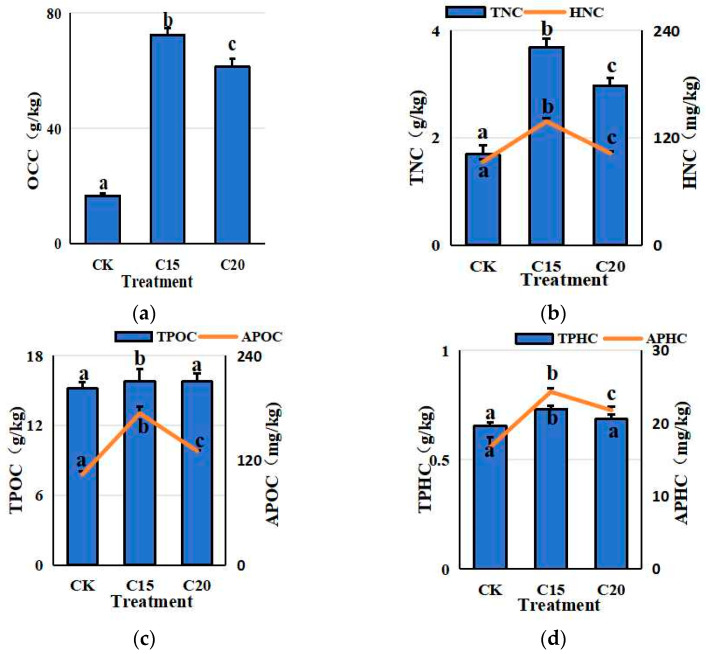
Nutritional element contents in rhizosphere soils of control and treatment groups. The OCC (**a**), TNC (**b**), HNC (**b**), TPOC (**c**), APOC (**c**), TPHC (**d**), and APHC (**d**) contents in rhizosphere soils. A significant difference between the data (*p* < 0.05) was indicated by bars with different letters.

The organic carbon contents (OCC), total nitrogen contents (TNC), total phosphorous contents (TPHC), total potassium contents (TPOC), hydrolysable nitrogen contents (HNC), available phosphorous contents (APHC), and available potassium contents (APOC) were 16.67–72.44 g/kg, 1.69–3.69 g/kg, 93.31–138.45 mg/kg, 15.18–15.84 g/kg, 103.88–173.96 mg/kg, 0.66–0.73 g/kg, and 16.74–24.32 g/kg, respectively (Figure 2). The two PGPR strain treatments significantly increased the OCC, TNC, HNC, APOC, and APHC in rhizosphere soil compared with the control (Figure 2)

### 2.3. Microbial Community in Tea Plant Rhizosphere Soil Irrigated with the PGPR Strains

High-throughput sequencing technology was used to investigate the microbial diversity in the rhizosphere of tea plants after the root irrigation experiments. The accession number was SAMN41050447, and the BioProject ID was PRJNA1103327.

**Figure 3:**
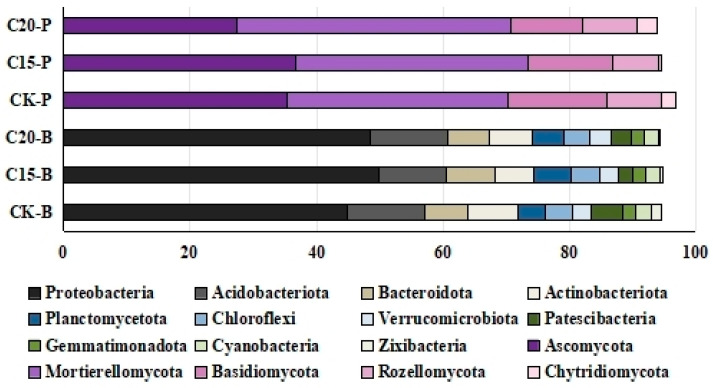
The distribution of microorganism with relative abundance greater than or equal to 1% in tea plant rhizosphere soil samples. Bacterial phyla (CK-B, C15-B, and C20-B) and fungal phyla (CK-P, C15-P, and C20-P) in soil.

There were 10 bacterial phyla (Proteobacteria, Acidobacteria, Bacteroidetes, Actinobacteria, Planctomycetes, Chlorofexi, Verrucomicrobia, Patescibacteria, Gemmatimonadota, and Cyanobacteria) with an average relative abundance of above 1% (Figure 3). Meanwhile, there were 11 bacterial phyla with an average relative abundance of above 1% in the CK samples (Figure 3). The relative abundances of Zixibacteria were 1.63% in the CK samples and lower than 1% in the rhizosphere samples of treated tea plants. Significant differences in bacterial diversity were found between the rhizosphere samples with different treatments. Proteobacteria, as the most prominent phyla, had a relative abundance of more than 40% in each sample (Figure 3). The relative abundances of Proteobacteria and Planctomycetota were higher in the soil samples treated with the PGPR strains than that in the CK samples, while the relative abundances of Actinobacteriota, Patescibacteria, and Zixibacteria were lower in the soil samples treated with the PGPR strains (Figure 3). 

Most of the fungal communities OTUs were classified as Ascomycota, Mortierellomycota, Basidiomycota, Rozellomycota, and Chytridiomycota (Figure 3). Ascomycota (with 35.37% of relative abundance) and Mortierellomycota (with 34.97% of relative abundance) were the prominent phyla in the CK soil samples, while Ascomycota (with 36.77% of relative abundance) and Mortierellomycota (with 36.67% of relative abundance) were the main phyla in the soil samples treated with strain C15. However, the relative abundance of Mortierellomycota (43.40%) showed that it was the main phyla in the soil samples treated with strain C20 (Figure 3). 

Alpha diversity indices (Shannon, Chao1, and Simpson) were calculated to compare with the microbial diversity in the tea plant rhizosphere of treated and CK soils. The fungal diversity was the highest in soil samples treated with strain C20 by the highest Simpson index, whereas the lowest was found in the CK soil samples (Table S1). There were no significant differences in the microbial diversities in treated and control tea plant rhizosphere samples according to the results of Shannon, Chao1, and Simpson (Table S1). 

**Figure 4:**
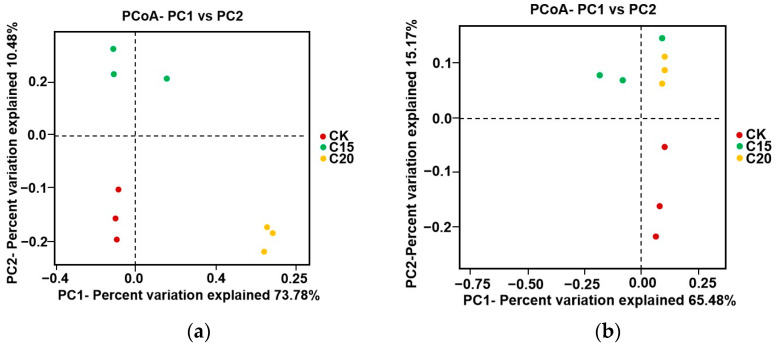
Differences in microorganism community structures in tea plant rhizospheres. Panels (**a**,**b**) show the differences in bacterial and fungal community structures, respectively.

A beta diversity analysis was used to evaluate the similarity of microbial compositions in the soil samples with different treatments. The species complexity at the three sampling sites were analyzed by PCoA (Figure 4). In the PCoA graph, the greater of the distance between the samples indicated the lower similarity between their communities. There was a considerable distance between strain-C15- and strain-C20-treated soil samples and CK soil samples in the graph (Figure 4). The result showed that the microbial compositions of these three soil samples were different from each other.

**Figure 5:**
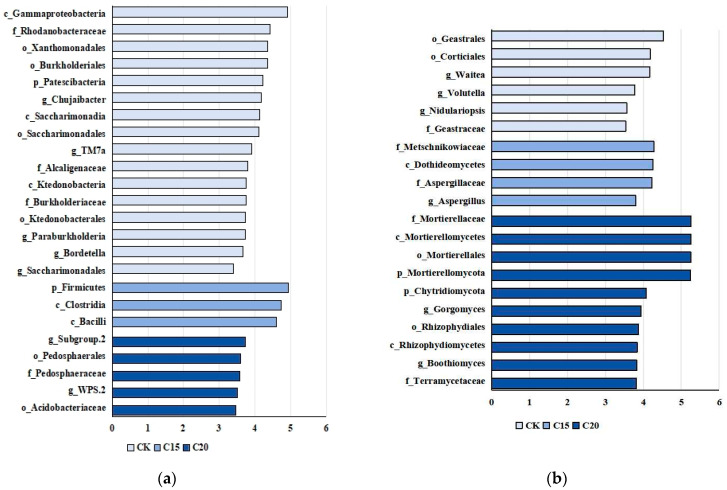
LEfSe analysis of microorganism for (**a**) differential enrichments of the bacterial features; and (**b**) fungi in tea plant rhizospheres.

Twenty-four distinct bacterial biomarkers and twenty distinct fungal biomarkers were identified via LDA (Figure 5). The strain C15 treatment enriched the phylotypes belonging to Firmicutes and Bacillota, while Acidobacteriota (Acidobacteriaceae) and Verrucomicrobiota accounted for the majority of rhizosphere bacteria in the soil samples treated with strain C20 (Figure 5a). The rhizosphere bacteria of CK sample contained an abundance of Proteobacteria and Patescibacteria phylum (Figure 5a). The fungi in rhizospheres were Ascomycota, Mortierellomycota, Basidiomycota, and Chytridiomycota (Figure 5b).

### 2.4. Metabolite Profiles of Tea Plant Root Irrigated with the PGPR Strains

**Figure 6:**
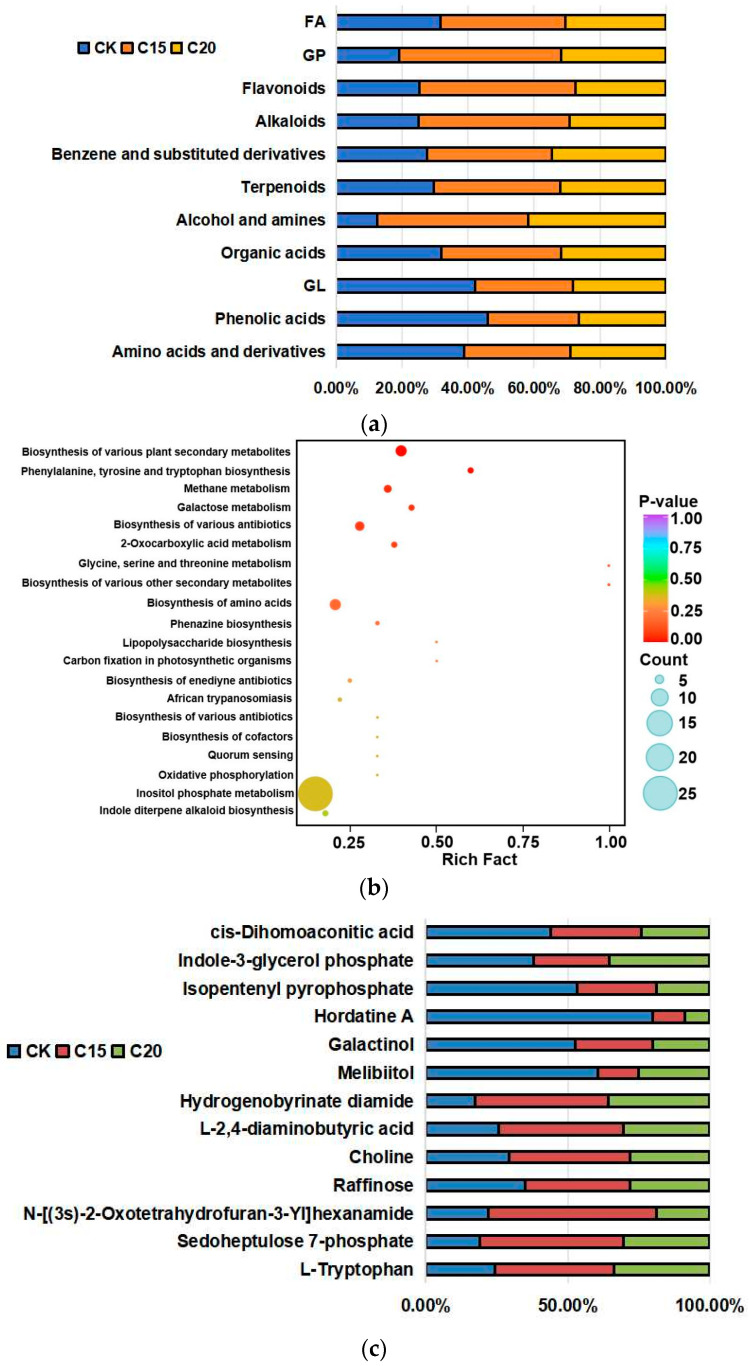
Data analysis and classification of identified metabolites in tea plant roots via HPLC-MS. (**a**) Classification of significantly different metabolites. (**b**) Top 20 pathways of upregulated and downregulated metabolites and DAMs on KEGG (rich factor on the X-axis and pathway on the Y-axis). The size of bubble indicates the number of involved DAMs. The color of bubble represents the degree of pathway enrichment. (**c**) DAMs’ up- and downregulation in various treatment groups.

A total of 3316 metabolites were detected in the CK, C15, and C20 treatments. Compared with CK, there were 483 metabolites, belonging to 11 metabolite groups (fatty acyl (FA), glyceryl phosphatide (GP), flavonoids, alkaloids, benzene and substituted derivatives, terpenoids, alcohol and amines, organic acids, glycerolipid (GL), phenolic acids, and amino acids and derivatives), showing a significant difference between the C15 and C20 treatments. The PGPR strain treatments significantly affected the metabolite contents in tea roots (Figure 6a). The relative abundances of GL, phenolic acids, and amino acids and derivatives were significantly lower in the tea plant roots in the C15 and C20 treatments compared with those in CK (Figure 6a). Other metabolites were significantly upregulated in the two treatments (Figure 6a). The results of PCA and OPLS-DA showed that there were significantly different metabolite profiles in the CK, C15, and C20 treatment as well (Figure S3).

The top 20 pathways with the highest enrichment in each group were selected by enrichment analysis and topological analysis to directly analyze the differences in metabolic pathways between the groups (Figure 6b). The KEGG pathway of differentially accumulated metabolites (DAMs) in the CK vs C15 and C20 treatments was highly enhanced in the “biosynthesis of various plant secondary metabolites”, “phenylalanine, tyrosine, and tryptophan biosynthesis”, “galactose metabolism”, “2-oxocarboxylic acid metabolism”, and the “biosynthesis of amino acids” listed in Figure 6b. This result was consistent with the results of the major differential metabolites obtained in this study.

Compared with the control group, the relative abundance of significantly different metabolites in the top 20 pathways are shown in Figure 6c. There were 13 DAMs in the top 20 pathways (Figure 6c). L-Tryptophan, sedoheputulose 7-phosphate, N-[(3s)-2-oxotetrahydrofuran-3-Yl] hexanamide, raffinose, choline, L-2,4-diaminobutyric acid, and hydrogenobyrinate diamide were upregulated in the tea plant roots treated by the C15 and C20 strains (Figure 6c). 

### 2.5. Correlations between the Strains with DAMs of Tea Plant Root, Rhizosphere Microenvironmental Factors, and Physiological Indices of Tea Plants

There was a greatest correlation between the 23 DAMs in tea root samples with the phosphorus solubilization and auxin production capacities of PGPR strains (Table S2). The Patescibacteria had a correlation with phosphorus solubilization and auxin production. The Chl concentration and plant height did not show significant correlations with the phosphorus solubilization and auxin production capacities of PGPR strains (Table S2). The rhizosphere soil element contents, except for TPOC, had a highly significant correlation with the phosphorus solubilization and auxin production capacities of PGPR strains (Table S2). 

Pearson’s correlation coefficient was used to determine the relationships between the DAMs in tea root samples, plant height, rhizosphere microbial diversity, and rhizosphere soil element contents. The factors that were significantly or highly significantly correlated with the DAMs are shown in Table S3. Proteobacteria, Actinobacteriota, Planctomycetota, Patescibacteria, and Basidiomycota had a highly significant correlation or significant correlation with the DAMs in tea root samples (Table S3). The rhizosphere soil element contents, except for TPOC, had the greatest correlation with the DAMs in tea root samples and Planctomycetota phyla (Table S3). Eight main principal components found in the results of PCA (Table S4) were the auxin production of the strains (0.928), with L-tryptophan (0.911), adenylosuccinic acid (0.896), sedoheptulose 7-phosphate (0.867), melibiitol (−0.924), 5-formyl-5,6,7,8-tetrahydromethanopterin (−0.883), Gramine (0.906), hydrogenobyrinate diamide (0.976), matairesinol (0.934), patescibacteria (−0.983), OCC (0.952), TNC (0.990), HNC (0.859), APOC (0.920), TPHC (0.870), and APHC (0.911) as the first principal components. Cyanobacteria (0.858) was a determining factor in the fifth principal component.

## 3. Materials and Methods

### 3.1. Soil Sample Collection 

Rhizosphere soil samples of tea plants were collected in April 2023 by the method described in a previous study [9] in Mianxian (32°58′46″ N, 106°42′25″ E), which is on the south side of Qinling Mountains in China. The altitude of the sampling site is 795.2 m. The depth of the collected soil was 10 to 20 cm under the ground surface surrounding the tea plant trunks. A total of 30 samples of rhizosphere soil were collected, mixed homogeneously, and stored at 4 °C before use.

### 3.2. Isolation of Plant-Growth-Promoting Bacteria (PGPR) 

A 250 mL sterilized flask with 50 mL of sterilized distilled water was prepared and a 5 g soil sample was added, and then incubated for 30 min with a shaking rate of 150 rpm. The 5 mL soil suspension was serially diluted to 10^−4^ dilution with sterile distilled water. Next, 100 µL of the 10^−4^ dilution was spread on beef extract–peptone agar plates. After incubation for 2 days, isolated strains with different morphologies were formed and further purified on the beef extract–peptone agar plates using the streak method. The purified isolated strains were named C1-n. 

The isolated strains were inoculated on inorganic phosphorus agar medium (HB8670; Hope BioTech, Jinan, China) for 4 days. The experiment was repeated in triplicate for each strain. Strains that had a soluble phosphorus circle were selected as PGPR strains.

Each PGPR strain was inoculated into a liquid inorganic phosphorus bacteria medium (HB8670-1; Hope BioTech, Jinan, China) and cultured for 4 days; the supernatant was collected from the liquid medium, which was centrifuged at 4 °C at 6000× *g* for 10 min. The soluble phosphorus content in the supernatant, used as the index to consider the phosphorus-solubilizing capability of PGPR, was determined by the molybdenum blue method [10].

Every PGPR strain was inoculated into the liquid beef extract–peptone medium with 100 mg/L L-Tryptophan (T0011; Solarbio, Beijing, China). The liquid medium was centrifuged at 4 °C at 6000× *g* for 10 min after incubation for 4 days. The Salkowski colorimetric method was used to evaluate the amount of auxin in the supernatant [11].

Each PGPR strain was inoculated into silicate bacteria agar medium (HB8548; Solarbio, Beijing, China) and A Sugai’s medium (HB8540; Solarbio, Beijing, China). After incubation for 4 days, the colony diameters in the two different media were recorded to evaluate the silicate dissolution and nitrogen fixation of the PGPR strains. All media used in experiments were cultured at 28 °C.

### 3.3. The 16S rDNA Sequencing of the PGPR Strains 

According to the manufacturer’s instructions (TIANamp Bacteria DNA Kit, DP302; Tiangen BioTech, Beijing, China), the genomic DNA of all the PGPR strains was extracted; then, 16S rDNA genes were amplified by the primers 27F (5′-AGA GTT TGA TCC TGG CTC AG-3′) and 1492R (5′-GGT TAC CTT GTT ACG ACT T-3′) (Invitrogen, Carlsbad, CA, USA). The PCR reaction solution constituent and PCR thermocycling program referred to the content of Wang et al. [11]. The 16S DNA PCR products were delivered and sequenced by Sangon Biotech, Shanghai, China. The nucleotide sequences of 16S rDNA were obtained and aligned into consensus sequences using Mega-11 software. Then, sequences of 16S rDNA were compared in the GenBank database (https://blast.ncbi.nlm.nih.gov/Blast.cgi, accessed on 23 April 2024). The sequence with longest length and 100% similarity of PGPR strains in each species was selected and submitted to the NCBI (https://www.ncbi.nlm.nih.gov/, accessed on 23 April 2024) website to obtain the GenBank accession number.

### 3.4. Plant Root Irrigation 

Tea plants (2-year-old), planted in same tea plant garden in which the soil samples were collected in Section 3.1 with similar height, were chosen for the root irrigation experiment. All plants were grown at 25 °C under continuous illumination (~1500 Lx). The period of irrigation treatment lasted for 42 d. Two PGPR strains with better performance were chosen for the root irrigation experiment. The 5 mL liquid beef extract peptone medium with which we had incubated each of the PGPR strains for 2 days at 28 °C was diluted to a final volume of 50 mL using sterilized water. These diluted liquid beef extract peptone media were used to irrigate twenty tea plants every 7 days for five times. For preparing a control group (CK), another twenty plants were irrigated by 50 mL of sterilized liquid beef extract peptone dilution medium instead.

### 3.5. Collection of Soil and Plant Root Samples and Determination of Tea Plant Physiological Indices 

The distance from the base of plant to the tip of plant main shoot of every tea plant was measured as plant height on day 7 after the irrigation experiment described above [12]. Then, the plant height of each treatment or control group was expressed by the mean and standard derivation calculated from the plant heights of twenty tea plants in each group. 

The eighth leaf of every plant was cut off with sterilized scissors and placed in a sterilized centrifuge tube. After it was weighed, it was ground in liquid nitrogen. Ethanol (95%) was used to extract the chlorophyll (Chl) in sample. The concentration of total chlorophyll in each group was calculated according to a previous study [13] and expressed by the mean and standard derivation of the results from twenty tea plants in the group. 

After the leaves were harvested, the rhizosphere soil samples in each group were collected and fully mixed. Then, they were stored at 4 °C before use. 

Then, all the plants were carefully removed from soil. The roots of plants were washed with distilled water. The cleaned plant roots of each group were cut and frozen by liquid nitrogen. 

### 3.6. Determination of Soil Element Contents

The element contents in soil samples which were collected on day 7 after the irrigation experiment were analyzed. The organic carbon contents (OCC), total nitrogen contents (TNC), total phosphorous contents (TPHC), total potassium contents (TPOC), hydrolysable nitrogen contents (HNC), available phosphorous contents (APHC), and available potassium contents (APOC) in soil samples were determined using the methods reported in Wang et al. [14]. 

### 3.7. Analysis of Microbial DNA Sequences in Soil Sample

The high-throughput amplicon sequencing technology was used to analysis the DNA sequences of microbes in soil samples. First, a DNA extraction kit (Fast DNA Spin Kit for Soil, MP Biomedicals, Santa Ana, CA, USA) was used to extract the genomes of microorganisms in soil samples. 515F/806R and ITS3-F/ITS4R (Invitrogen, Carlsbad, CA, USA) were used as the specific primers to amplify the 16S rRNA genes of the V4 regions in soil bacteria and ITS4 regions in soil fungus, respectively. The procedure of PCR reaction was carried out using the same method in Wang et al. [12]. The lengths and concentrations of PCR products were detected by gel electrophoresis. The products with concentrations at 290–310 bp length for the 16S or 370–450 bp length for the ITS were mixed at the same density ratio and purified by the EZNA Gel Extraction Kit (D2500-02, Omega Bio-Tek, Norcross, GA, USA). The NEBNext Ultra DNA Library Prep Kits for Illumina (New England Biolabs, Ipswich, MA, USA) were used to set up the DNA libraries. The final sequences were output by an Illumina HiSeq 2500 platform (Illumina, CA, USA). After we removed the chimera sequence and singleton OTU, the assigned operational taxonomic unit (OTU) to represent a species was analyzed by carrying out the statistics of paired-end raw reads. The taxonomic information was annotated by the Silva database (https://www.arb-silva.de/, accessed on 2 May 2024, Max Planck Society, Gottingen, Federal Republic of Germany). Then, the information table of OTU taxonomy synthesis was obtained and submitted to the Sequence Read Archive (https://submit.ncbi.nlm.nih.gov/subs/sra/, accessed on 2 May 2024, NCBI, Bethesda, MD, USA). 

Alpha diversity indices were calculated using QIIME2 (https://qiime2.org/, accessed on 2 May 2024, Northern Arizona University, San Francisco Mountains, AZ, USA) and expressed as mean and standard error. Significant differences between different species were determined via linear discriminant analysis (LDA). The effect size (LEfSe) (https://github.com/biobakery/galaxy_lefse, accessed on 2 May 2024, Harvard University, Cambridge, MA, USA) of two was used as the default setting value for the LDA score. Bray–Curtis and weighted and unweighted UniFrac for beta diversity were calculated using QIIME2 (https://qiime2.org/, accessed on 2 May 2024, Northern Arizona University, San Francisco Mountains, AZ, USA) and displayed in R software (V2.15.3). The qiime2 and ggplot2 packages in R software (V3.2.0) were used to visualize the results of principal coordinates analysis (PCoA).

### 3.8. Metabolomics Analysis of Tea Plant Root Sample

The roots of tea plants were homogenized by a grinder (30 HZ) for 20 s. An aliquot of 400 μL of solution (Methanol: Water = 7:3, *V*/*V*) containing internal standard was added into 20 mg of the ground sample and shaken at 1500 rpm for 5 min. After it was placed in ice bath for 15 min, the sample was centrifuged at 12000 rpm for 10 min (4 °C). An aliquot of 300 μL of the supernatant was collected and placed in −20 °C for 30 min. The sample was then centrifuged at 12,000 rpm for 3 min (4 °C). The supernatant (100 μL) was analyzed using positive ion condition and eluted from T3 column (Waters ACQUITY Premier HSS T3 Column 1.8 µm, 2.1 mm × 100 mm) using 0.1% formic acid in water as solvent A and 0.1% formic acid in acetonitrile as solvent B. The gradient for solvent B was as follows: 5 to 20% in 2 min, increased to 60% in the following 3 min, increased to 99% in 1 min and held for 1.5 min, then decreased to 5% mobile phase B within 0.1 min, and held for 2.4 min. The data acquisition was operated using the information-dependent acquisition (IDA) mode using Analyst TF 1.7.1 Software (Sciex, Concord, ON, Canada). The original data file was converted into mzXML format by ProteoWizard software (3.0.9134). The peak extraction, peak alignment, and retention time correction were performed by XCMS program. The “SVR” method was used to correct peak area. The peaks with detection rate lower than 50% in each group of samples were not used. After that, the metabolic identification was obtained by searching the built database, integrated public database, AI database, and met DNA.

The unsupervised principal component analysis (PCA) was performed by the function of prcomp within R (www.r-project.org, accessed on 24 July 2024, University of Auckland, Auckland, New Zealand). The data was unit-variance-scaled before unsupervised PCA. For the orthogonal partial least squares–discriminant analysis (OPLS-DA), differential metabolites were determined by VIP (VIP > 1) and *p*-value value < 0.05, Student’s *t* test). VIP values were extracted from the OPLS-DA result which contained score plots, and permutation plots was generated using R package MetaboAnalystR. Significantly different metabolites were screened based on variable importance in projection (VIP) obtained by the OPLS-DA model and *p*-value obtained by univariate analysis. Identified metabolites were annotated using the Kyoto Encyclopedia of Genes and Genomes (KEGG) compound database (http://www.kegg.jp/kegg/compound, accessed on 24 July 2024, Institute of Chemistry, Kyoto University, Tokyo, Japan), The annotated metabolites were then mapped to the KEGG Pathway database (http://www.kegg.jp/kegg/pathway.html, accessed on 24 July 2024, Institute of Chemistry, Kyoto University, Tokyo, Japan). Significantly enriched pathways were identified with hypergeometric test’s *p*-value for the list of metabolites. 

### 3.9. Data Analysis

All experiments were repeated in triplicate. The mean and standard derivation were used to express the results of phosphorus and auxin dissolution of PGPR strains, colony diameters of PGPR strains in silicate bacteria media and A Sugai’s medium, and nutritive element contents in tea plant rhizospheres. 

The nature of the links between the phosphorus solubilizing ability and auxin production ability of PGPR strains with differential metabolites in roots of tea plants, rhizosphere soil microbial diversity, plant height, leaf chlorophyll, and soil element content was identified by Pearson’s correlation coefficients [15,16,17]. The correlations between differential metabolites in roots of tea plants with plant height, rhizosphere soil microbial diversity, and soil element contents, and the correlation of soil element contents with rhizosphere soil microbial diversity were also analyzed. The nature of the links between phosphorus solubilizing ability, auxin production ability of PGPR strain with rhizosphere microenvironmental factors, plant growth status, and differential metabolites in root of tea plant was analyzed by principal component analysis (PCA) using SPSS v23.0 software [18].

## 4. Discussion

### 4.1. The PGPR Strains in Rhizosphere Soil of Tea Plant

The PGPRs are a group of soil bacteria having the function of promoting plant growth through the most important and direct mechanisms in soil such as the solubilization of nutrients, nitrogen fixation, the production of growth regulators, etc. [19]. Based on these capabilities which were evaluated in the cultural experiments of this study, a total of 38 PGPR strains, belonging to *Acinetobacter* sp., *Erwinia* sp., *Kluyvera* sp., *Leclercia* sp., *Pantoea* sp., *Rouxiella* sp., and *Serratia* sp., were isolated from the collected soil samples. Different PGPR strains showed different inorganic phosphate-solubilizing, silicate-solubilizing, and auxin-production capabilities. 

*Pseudomonas*, *Acinetobacter*, *Erwinia*, and *Pantoea* are the important PGPR strains with better capacities and exhibited a significant improvement in major plant growth parameters like the germination percentage and root structure [20]. Genus *Kluyvera* was the PGPR strain that decreased heavy metal toxicity in plants [21]. The bacteria identified as *Leclercia* had a high auxin production and strong salinity stress tolerance [22]. *Rouxiella* and *Serratia* were discovered as novel biocontrol agents of fungal pathogens [23]. According to our results and previous similar studies, the PGPR isolated from tea plant rhizosphere soil could enrich and fertilize the nutrients available by tea plant and help tea plant to grow well and resist various pathogens [12].

### 4.2. Effects of the PGPR Strains in Root Irrigation on Plant Growth and Soil Element Contents

The selected strains C15 and C20 belonging to the *Erwinia* and *Serratia* genus, respectively, had a higher plant height in the treated tea plants. It was reported that carbon, nitrogen, phosphorus, and potassium in soil play an important role in terrestrial ecosystems by affecting the soil microbial movement, litter decomposition, and nutrient cycling and accumulation [24]. The concentrations of major environmental elements in the treatments were determined (Figure 2). The PGPR strains significantly increased the OCC, TNC, HNC, APOC, and APHC in rhizosphere soil compared with the control soil. Organic carbon is the major nutrient responsible for the physical, chemical, and biological characteristics of soil [25]. It significantly affects the development and production of the plant [26]. Both TNC and HNC are important indicators of soil nitrogen content and soil fertility [15]. Soil TPOC and APOC are closely related to plant growth and crop yield [27]. Phosphorus (P), as an essential macronutrient for plants, plays a crucial role in the synthesis of nucleic acids, enzymes, and coenzymes [28]. P exists in organic (Po) and inorganic (Pi) forms in soil, while only the inorganic orthophosphate anions (PO_4_^3−^) could be accepted by plants [29]. Insoluble phosphorus elements could be solubilized by secreting organic acids, accessed by PGPRs. The phosphatases, such as acid phosphatase (ACP), alkaline phosphatase (ALP), and phytase, could be released by the PGPR strains to mineralize Po [30]. The selected PGPR strains significantly increased the TPHC and APOC in the rhizosphere soil of tea plants in this study. 

### 4.3. Microbial Diversity in Tea Plant Rhizosphere Soil Irrigated with the PGPR Strains

The PGPR strains could improve the microbial community structure and sustainable soil cultivation [31,32]. In this study, the microbial diversity in the rhizosphere of tea plants after the root irrigation by PGPR strains were investigated.

Proteobacteria, Acidobacteria, Bacteroidetes, Actinobacteria, Planctomycetes, Chlorofexi, Verrucomicrobia, Patescibacteria, Gemmatimonadota, and Cyanobacteria were found as the main bacterial phyla in soil samples of the PGPR strain treatments. Meanwhile, there were 11 main bacterial phyla in the CK samples. These bacterial phyla with an average relative abundance of above 1% were the common bacterial phyla in plant rhizosphere soil [33]. The fungal diversity of plant rhizosphere soil was also affected under the action of growth-promoting bacteria [8]. Soil is one of the major habitats of bacteria and fungi; their interactions are part of a communication network that keeps microhabitats in balance [34]. Through this interaction, bacterial diversity also directly affects fungal diversity in the same area [35]. *Erwinia* and *Serratia*, as two of the most important functional groups in bacterial microbiota, could interact with soil fungal communities to affect soil microbial diversity [36,37].

The rhizosphere is the central hotspot of nutrient uptake by plants, microbial activities, and plant–soil–microbial interactions [38]. Moreover, rhizosphere soil microbial diversity is very important for plant growth and the stability of the rhizosphere microenvironment [39]. These changes found in the tea plant rhizosphere soil treated with the PGPR strains in this study might highlight a direct or indirect method of increasing crop yields and promoting plant growth [40]. 

### 4.4. Metabolite Profiles of Tea Plant Root Irrigated with the PGPR Strains

Compared with CK, there were 483 metabolites with a significant difference between the C15 and C20 treatments. The PGPR strain treatments significantly affected the metabolite contents in tea roots. GL, phenolic acids, and amino acids and derivatives were significantly different in the tea plant root metabolites of the CK, C15, and C20 treatments. Amino acids and derivatives are one of the major forms of nitrogen in plants and play an important role in plant growth and defense [41]. Phenolic acids are aromatic secondary plant metabolites for plant defense against biotic and abiotic stresses [42]. GLs take part in the biosynthesis of galactolipids such as digalactosyldiacylglycerol, which was reported as an important component of plasma membranes of plants [43]. 

To analyze the differences in metabolic pathways between the groups, the top 20 pathways with the highest enrichment in each group were selected by enrichment analysis and topological analysis. The KEGG pathway of differentially accumulated metabolites (DAMs) in the CK vs C15 or C20 treatment was highly enriched in the “biosynthesis of various plant secondary metabolites”, “phenylalanine, tyrosine and tryptophan biosynthesis”, “galactose metabolism”, “2-oxocarboxylic acid metabolism”, and the “biosynthesis of amino acids”. 

Compared with the control group, L-tryptophan, sedoheputulose 7-phosphate, N-[(3s)-2-oxotetrahydrofuran-3-Yl] hexanamide, raffinose, choline, L-2,4-diaminobutyric acid, and hydrogenobyrinate diamide were upregulated in the tea plant root treated by the C15 and C20 strains. Tryptophan is one of aromatic amino acids (AAAs) that are used for the synthesis of proteins and serve as the precursors of numerous natural products, such as pigments, alkaloids, hormones, and cell wall components [44]. Sedoheputulose 7-phosphate is a member of the reduced pentose phosphate cycle in photosynthesis [45]. N-[(3s)-2-Oxotetrahydrofuran-3-Yl] hexanamide belong to the class of sensing signal molecules involved in distance signal transduction between bacteria and plants [46]. The raffinose family of oligosaccharides (RFOs) are α-1, 6-galactosyl extensions of sucrose with a galactosyl group donated by galactinol. The synthesis of galactinol is a key and absolute requirement for entering into the pathway of RFO biosynthesis [47]. Choline, as a precursor of glycine betaine (GB) and phospholipids, is known to play roles in the osmoticregulation of the cytoplasm [48].

### 4.5. Correlations Between the Strains with DAMs of Tea Plant Root, Rhizosphere Microenvironmental Factors, and Physiological Indices of Tea Plant

The soil environment, especially the rhizosphere, is interesting and complicated. There are so many interactions taking place in the soil; plants and microorganisms symbiotically mediate and/or catalyze the turnover of elements in rhizosphere soils. These interactions determine the properties of soil as a medium for the growth and activities of plants and soil microorganisms [49].

Patescibacteria was the only rhizosphere soil microorganism that had a correlation with auxin production. A highly significant correlation was found between the rhizosphere soil element contents, except for TPOC, and the phosphorus solubilization and auxin production capacities of the PGPR strains.

Proteobacteria, Actinobacteriota, Planctomycetota, Patescibacteria, and Basidiomycota had a highly significant correlation or significant correlation with the DAMs in the tea root samples. The rhizosphere soil element contents, except for TPOC, had the greatest correlation with the DAMs in the tea root samples and Planctomycetota phyla.

The metabolites secreted into the rhizosphere by roots are involved in several processes [50]. The plant–PGPR interaction, with a special reference to chemical signaling molecules, is not understood clearly [51]. Some studies have suggested that plant roots can adapt to the environment through changes in metabolites [52].

**Figure 7:**
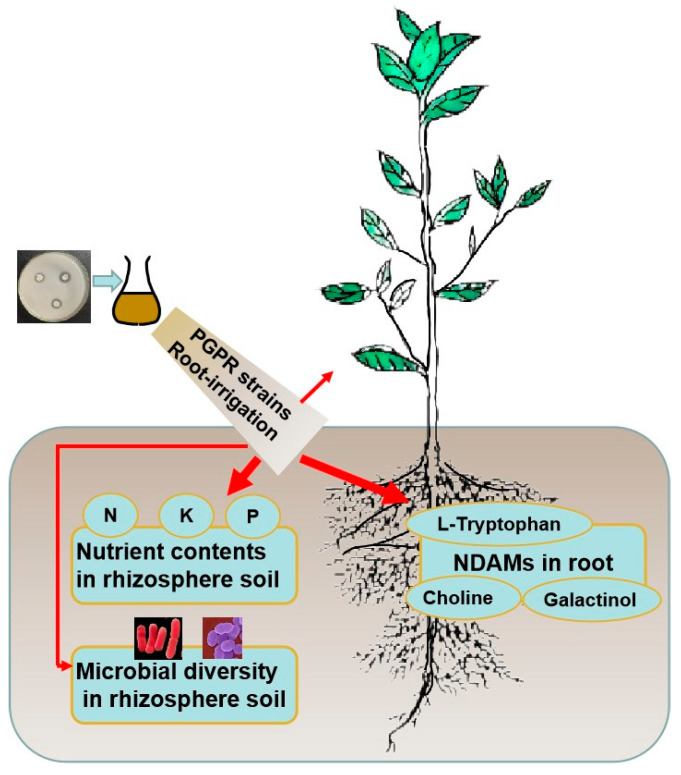
The relationship between the PGPR strains, rhizosphere microenvironmental factors, and tea plant growing. Notes: The red arrows indicate the correlations. The thicker the arrow line, the stronger the correlation.

In this study, there was a significant or extremely significant correlation between the root metabolites and PGPR strains. At the same time, the PGPR strains significantly changed the nutrient contents in rhizosphere soil after the plants were irrigated by the strains. The root irrigation had the effect on the rhizosphere microbial community structure. The rhizosphere nutritional elements also showed a significant or extremely significant correlation with the root metabolites. According to the results, the contents of nutritional elements in tea rhizosphere soil were significantly increased by the PGPR strains (Figure 7). The root metabolites that were involved in amino acid synthesis, matter transport, etc. were enhanced by the interaction between the PGPR strains and tea plants (Figure 7). The PGPR strains had a significant effect on the diversity of rhizosphere microorganisms (Figure 7).

## 5. Conclusions

A total of 38 PGPR strains were isolated and evaluated in this study. Based on their sequences, they were the phyla Proteobacteria and classified into *Acinetobacter*, *Erwinia*, *Kluyvera*, *Leclercia*, *Pantoea*, *Pseudomonas*, *Rouxiella*, and *Serratia*. The capabilities of inorganic phosphate-solubilizing, auxin production, silicate-solubilizing activity, and nitrogen fixation were determined in the different strains. The selected strains C15 and C20 PGPR treatments had a higher Chl concentration and plant height in the treated tea plants. The irrigation with PGPR strains also significantly increased OCC, TNC, HNC, TPOC, APOC, TPHC, and APHC in the rhizosphere soil compared with the control. There were significant differences in the microbial diversity and metabolite profile between the rhizosphere samples in the treatment and control groups. The contents of nutrients in tea rhizosphere soil were significantly increased by the irrigation of PGPR strains. The root metabolites were enhanced by the interaction between the PGPR and tea plant. Overall, the selected PGPR strains improved the diversity of rhizosphere microorganisms and increased the concentration of the required nutrients for tea plant growing.

## Figures and Tables

**Table 1:** Classifications of identified PGPR strains.

Scheme	Accession Number	Sequence Length (bp)	Related Type Strain	Type Strain Name	NCBI Taxonomy ID	Similarity to Type Strain (%)	The Number of Strains Belonging to Same Genus
C38	OR234767	1112	*Acinetobacter calcoaceticus*	DSM 30006T	981331	99%	1
C2	OR234768	1127	*Acinetobacter lwoffii*	DSM 2403T	28090	99%	1
C15	OR234769	949	*Erwinia* sp.	-	-	-	6
C42	OR234770	1163	*Erwinia billingiae*	DSM 17872T	182337	99%	1
C21	OR234771	845	*Kluyvera intermedia*	DSM 4581T	61648	99%	1
C1	OR234772	1207	*Leclercia* sp.	-	-	-	2
C11	OR234773	1185	*Pantoea* sp.	-	-	-	3
C39	OR234774	1042	*Pseudomonas* sp.	-	-	99%	7
C26	OR234775	1118	*Pseudomonas cedrina*	DSM 17516T	651740	99%	1
C77	OR234776	1150	*Pseudomonas jessenii*	DSM 17150T	77298	99%	1
C79	OR234777	1086	*Pseudomonas moorei*	DSM 12647T	395599	-	3
C71	OR234778	1196	*Pseudomonas putida*	DSM 291T	303	99%	6
C58	OR234779	1191	*Rouxiella* sp.	-	-	-	3
C20	OR234782	1020	*Serratia* sp.	-	-	-	2

## Data Availability

The data are contained within the article and Supplementary Materials.

## Supplementary Materials

The following supporting information can be downloaded at: https://www.mdpi.com/article/10.3390/plants13182659/s1, Figure S1: Phosphorus-solubilizing capabilities of isolated PGPR strains; Figure S2: Plant physiology of tea plants; Figure S3: Data analysis of identified metabolites in tea plant roots via HPLC-MS; Table S1: Alpha diversity index of bacteria (CK-B, C15-B, and C20-B) and fungi (CK-P, C15-P, and C20-P) in tea plant rhizospheres; Table S2: Pearson’s correlation analysis of phosphorus solubilizing ability and auxin production ability of PGPR strains with differential metabolites in roots of tea plants, rhizosphere soil microbial diversity, plant growth status, and soil element content; Table S3: Pearson’s correlation analysis of differential metabolites in roots of tea plants with plant height, rhizosphere soil microbial diversity, and soil element contents; Pearson’s correlation analysis of soil element contents with rhizosphere soil microbial diversity; Table S4: Eigenvector of eight principal components of phosphorus solubilizing ability and auxin production ability of PGPR strains, plant growth status, differential metabolites in roots of tea plants, and rhizosphere microenvironment factors.
