# Supplementary material for: Characterization of Plant-Growth-Promoting Rhizobacteria for Tea Plant (Camellia sinensis) Development and Soil Nutrient Enrichment
Source: Plants (Basel). 2024 Sep 23;13(18):2659. doi: 10.3390/plants13182659 (PMC11434996; doi:10.3390/plants13182659)
Supplement: Supplementary file 1 [file plants-13-02659-s001.zip › plants-3147830-supplementary.pdf]

plants
MDPI

Supplementary Materials

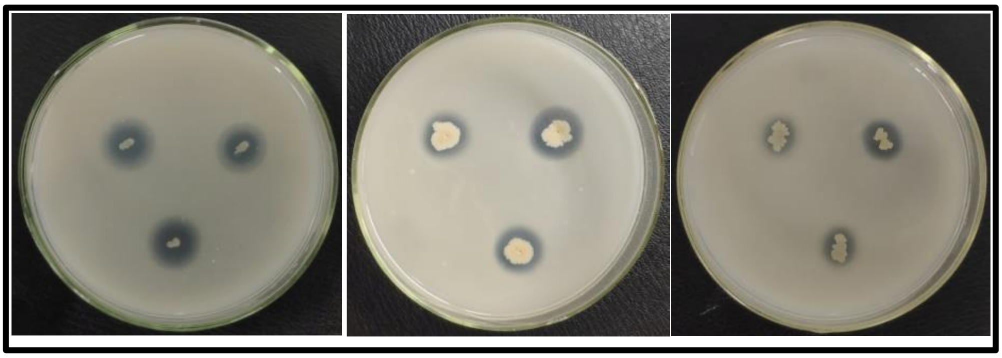

Figure S1. Phosphorus-solubilizing capabilities of PGPRs. Visible colonies on the inorganic phosphorus bacteria agar medium are showed in Figure S1.

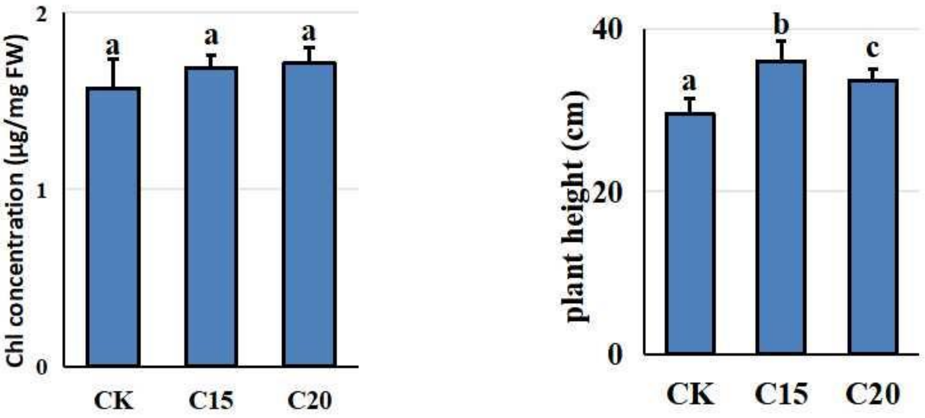

(**a**) (**b**)

Figure S2. Plant physiology of tea plants. Chl (Chlorophyll) concentration (a) and plant height (b) of tea plants. Significant difference between the data (p <0.05) was showed by bars with different letters .

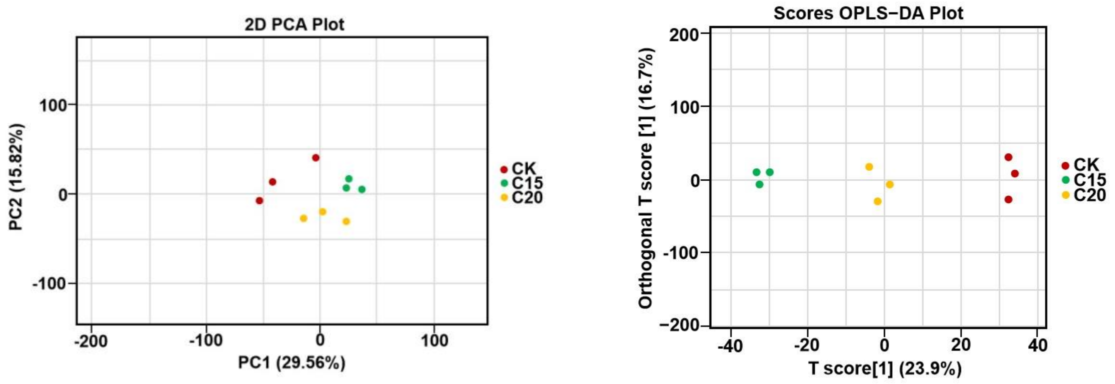

(**a**) (**b**)

Figure S3. Data analysis of identified metabolites in tea plant roots via HPLC-MS. (a) PCA score plot of PC1 vs PC2. (b) Orthogonal partial least squares-discriminant analysis (OPLS-DA) score plot.

Table S1. Alpha diversity index of microorganism in different soil samples

	Shannon	Chao	Simpson
CK-B	6.488±0.464a	6769.557±329.767a	0.010±0.006a
C15-B	6.760±0.067a	6718.293±298.701a	0.007±0.000a
C20-B	6.724±0.034a	7254.478±61.961a	0.007±0.000a
CK-P	3.361±0.293a	1153.860±35.807a	0.136±0.031a
C15-P	3.329±0.126a	1222.712±54.535a	0.137±0.024a
C20-P	3.239±0.282a	1121.653±65.726a	0.197±0.014b

Note: CK-B, C15-B, and C20-B referred to the bacteria in the three groups of treated soil samples, respectively. CK-P, C15-P and C20-P referred to the fungus.

Table S2. Pearson's correlation analysis of phosphorus solubilizing ability and auxin production ability of PGPR strains with differential metabolites in roots of tea plants, rhizosphere soil microbial diversity, plant growth status and soil nutrient element content.

Capacity of strains	$C_{11}H_{12}N_2O_2$	$C_{12}H_{23}N_2O_9PS$	$C_{17}H_{21}N_4O_9P$	$C_{14}H_{18}N_5O_{11}P$	$C_7H_{15}O_{10}P$	$C_{10}H_{17}NO_3$	$C_{18}H_{32}O_{16}$	$C_{12}H_{24}O_{11}$
Phosphorus	0.719*	0.315	0.739*	0.627	**0.805****	**0.932****	-0.621	-0.300
Auxin	0.754*	**0.802****	0.647	0.762*	0.670*	0.518	**-0.820****	-0.762*
	$C_{12}H_{22}O_{11}$	$C_{24}H_{42}O_{21}$	$C_{31}H_{45}N_6O_{17}P$	$C_{11}H_{14}N_2$	$C_{28}H_{38}N_8O_4$	$C_5H_{14}NO^+$	$C_5H_{12}O_7P_2$	$C_{11}H_{14}NO_6P$
Phosphorus	**0.941****	**0.872****	-0.562	0.629	-0.458	**0.815****	-0.316	**-0.835****
Auxin	0.490	0.502	**-0.855****	**0.847****	**-0.839****	0.449	**-0.879****	-0.646
	$C_8H_{10}O_6$	$C_{10}H_{10}O_6$	$C_4H_{10}N_2O_2$	$C_{45}H_{62}N_6O_{12}$	$C_{12}H_{24}O_{17}P_2$	$C_{18}H_{23}N_5O_6$	$C_{20}H_{22}O_6$	-
Phosphorus	-0.182	0.647	0.782*	0.790*	0.339	**0.861****	0.692*	-
Auxin	**-0.840****	**0.884****	0.593	**0.912****	**0.813****	0.676*	**0.990****	-
	Proteobacteria	Acidobacteriota	Bacteroidota	Actinobacteriota	Planctomycetota	Chloroflexi	Verrucomicrobiota	Patescibacteria
Phosphorus	0.546	-0.492	0.525	-0.478	0.532	0.558	-0.113	-0.761*
Auxin	0.672*	-0.288	0.276	-.584	0.431	.251	0.127	**-0.918****
	Gemmatimonadota	Cyanobacteria	Zixibacteria	Ascomycota	Mortierellomycota	Basidiomycota	Rozellomycota	Chytridiomycota
Phosphorus	-0.104	-0.029	-0.224	0.341	-0.130	-0.089	-0.136	-0.456
Auxin	0.014	0.010	-0.451	-0.173	0.332	-0.546	-0.091	-0.104
	OCC	TNC	HNC	TPOC	APOC	TPHC	APHC	-
Phosphorus	0.703*	**0.814****	**0.985****	0.319	**0.928****	**0.882****	0.792*	-
Auxin	**0.980****	**0.960****	0.737*	0.469	**0.842****	**0.817****	**0.956****	-
	chl	Plant height	-	-	-	-	-	-
Phosphorus	0.258	0.553	-	-	-	-	-	-
Auxin	0.524	0.689*	-	-	-	-	-	-

Notes: Phosphorus, Phosphorus-solubilizing capacity of strains used for root irrigation; Auxin, Auxin production capacity of strains used for root irrigation; $C_{11}H_{12}N_2O_2$, L-Tryptophan; $C_{12}H_{23}N_2O_9PS$, N-[(R)-4-phosphopantothenoyl]-L-cysteine; $C_{17}H_{21}N_4O_9P$, Flavin mononucleotide; $C_{14}H_{18}N_5O_{11}P$, Adenylosuccinic acid; $C_7H_{15}O_{10}P$, Sedoheptulose 7-phosphate; $C_{10}H_{17}NO_3$, N-[(3s)-2-Oxotetrahydrofuran-3-Yl]hexanamide; $C_{18}H_{32}O_{16}$, Raffinose; $C_{12}H_{24}O_{11}$, Melibiitol; $C_{12}H_{22}O_{11}$, Galactinol; $C_{24}H_{42}O_{21}$, Stachyose; $C_{31}H_{45}N_6O_{17}P$, 5-Formyl-5,6,7,8-tetrahydromethanopterin; $C_{11}H_{14}N_2$, Gramine; $C_{28}H_{38}N_8O_4$, Hordatine A; $C_5H_{14}NO^+$, Choline; $C_5H_{12}O_7P_2$, Isopentenyl pyrophosphate; $C_{11}H_{14}NO_6P$, Indole-3-glycerol phosphate; $C_8H_{10}O_6$, cis-Dihomoaconitic acid,; $C_{10}H_{10}O_6$, Chorismic acid; $C_4H_{10}N_2O_2$, L-2,4-diaminobutyric acid; $C_{45}H_{62}N_6O_{12}$, Hydrogenobyrinate diamide; $C_{12}H_{24}O_{17}P_2$, bis(1L-myo-inositol) 3,1'-phosphate 1-phosphate; $C_{18}H_{23}N_5O_6$, 8-Demethyl-8-(dimethylamino)riboflavin; $C_{20}H_{22}O_6$, Matairesinol; The underline and bold entries were used to show highly significant correlation between the two data. * $p < 0.05$. ** $p < 0.01$.

Table S3. Pearson's correlation analysis of differential metabolites in roots of tea plants with plant height, rhizosphere soil microbial diversity and soil nutrient element contents, Pearson's correlation correlation analysis of soil element contents with rhizosphere soil microbial diversity.

	Plant height	Proteobacteria	Actinobacteriota	Planctomycetota	Patescibacteria	Basidiomycota	OCC	TNC	HNC	TPOC	APOC	TPHC	APHC
$C_{11}H_{12}N_2O_2$	0.792*	0.747*	**-0.799****	0.774*	**-0.892****	-0.286	**0.830****	**0.878****	0.747*	0.273	**0.830****	0.715*	0.737*
$C_{12}H_{23}N_2O_9PS$	0.706*	0.346	-0.656	0.423	-0.708*	-0.493	0.774*	0.742*	0.389	0.505	0.547	0.414	0.727*
$C_{17}H_{21}N_4O_9P$	0.639	0.455	-0.521	0.383	-0.760*	-0.234	0.733*	0.728*	**0.799****	-0.208	**0.849****	0.630	0.704*
$C_{14}H_{18}N_5O_{11}P$	0.748*	0.699*	**-0.901****	**0.852****	**-0.898****	-0.459	**0.831****	**0.864****	0.670*	0.360	0.752*	0.602	0.722*
$C_7H_{15}O_{10}P$	0.534	0.715*	-0.743*	**0.870****	**-0.842****	-0.364	0.774*	**0.825****	**0.843****	0.295	**0.821****	0.688*	0.719*
$C_{10}H_{17}NO_3$	0.444	0.680*	-0.445	0.503	-0.659	-0.062	0.499	0.664	0.786*	0.304	0.721*	**0.860****	0.540
$C_{18}H_{32}O_{16}$	0.352	0.466	-0.416	0.611	-0.616	-0.042	0.540	0.690*	**0.888****	0.489	0.770*	0.785*	0.658
$C_{12}H_{24}O_{11}$	-0.653	**-0.896****	**0.799****	-0.767*	**0.923****	0.552	**-0.857****	**-0.884****	-0.649	-0.407	-0.728*	-0.735*	-0.727*
$C_{12}H_{22}O_{11}$	-0.606	-0.784*	0.720*	-0.509	**0.824****	0.694*	-0.765*	-0.733*	-0.346	-0.139	-0.504	-0.529	-0.568
$C_{24}H_{42}O_{21}$	0.428	0.364	-0.377	0.465	-0.572	0.055	0.501	0.648	**0.894****	0.444	0.789*	0.761*	0.652
$C_{31}H_{45}N_6O_{17}P$	-0.713*	-0.753*	**0.815****	-0.653	**0.875****	0.585	**-0.853****	**-0.858****	-0.603	-0.646	-0.698*	-0.648	-0.775*
$C_{11}H_{14}N_2$	0.644	0.715*	-0.769*	0.675*	**-0.938****	-0.596	**0.863****	**0.901****	0.659	0.206	0.744*	0.722*	0.759*
$C_{28}H_{38}N_8O_4$	-0.506	-0.459	0.220	-0.236	0.649	0.263	**-0.835****	-0.774*	-0.548	-0.387	-0.667*	-0.667*	**-0.832****
$C_5H_{14}NO^+$	0.593	0.596	-0.661	0.747*	-0.685*	-0.007	0.516	0.671*	0.759*	0.210	0.728*	0.685*	0.505
$C_5H_{12}O_7P_2$	-0.645	-0.618	0.464	-0.269	0.743*	0.498	**-0.836****	-0.779*	-0.382	-0.410	-0.566	-0.614	-0.744*
$C_{11}H_{14}NO_6P$	-0.448	-0.400	0.149	-0.335	0.564	-0.133	-0.670*	-0.728*	**-0.853****	-0.477	**-0.826****	**-0.820****	**-0.802****
$C_8H_{10}O_6$	-0.531	-0.530	0.367	-0.133	0.649	0.574	-0.775*	-0.684*	-0.262	-0.422	-0.448	-0.508	-0.689*
$C_{10}H_{10}O_6$	0.582	0.644	-0.328	0.161	**-0.804****	-0.394	**0.852****	**0.838****	0.687*	0.141	0.788*	**0.859****	**0.833****
$C_4H_{10}N_2O_2$	0.324	0.580	-0.384	0.390	-0.714*	-0.361	0.650	0.676*	**0.810****	-0.123	0.767*	0.724*	0.640
$C_{45}H_{62}N_6O_{12}$	0.763*	0.748*	-0.782*	0.618	**-0.993****	-0.526	**0.931****	**0.966****	**0.823****	0.277	**0.899****	**0.828****	**0.872****
$C_{12}H_{24}O_{17}P_2$	0.353	0.663	-0.637	0.517	-0.792*	**-0.883****	0.765*	0.748*	0.387	0.420	0.462	0.546	0.654
$C_{18}H_{23}N_5O_6$	0.628	0.300	-0.620	0.595	-0.769*	-0.192	0.721*	**0.809****	**0.882****	0.191	**0.871****	0.672*	0.789*
$C_{20}H_{22}O_6$	0.678*	0.737*	-0.572	0.433	**-0.912****	-0.525	**0.966****	**0.954****	0.746*	0.536	**0.841****	**0.854****	**0.944****
OCC	0.689*	-0.316	0.533	**-0.944****	-0.521	0.689*	-	-	-	-	-	-	-
TNC	0.716*	-0.407	0.608	**-0.969****	-0.466	0.716*	-	-	-	-	-	-	-
HNC	0.540	-0.419	0.549	**-0.802****	-0.154	0.540	-	-	-	-	-	-	-
TPOC	0.323	-0.043	0.303	-0.316	-0.174	0.323	-	-	-	-	-	-	-
APOC	0.598	-0.442	0.507	**-0.876****	-0.188	0.598	-	-	-	-	-	-	-
TPHC	0.739*	-0.652	0.374	**-0.808****	-0.214	0.739*	-	-	-	-	-	-	-

Notes: $C_{11}H_{12}N_2O_2$, L-Tryptophan; $C_{12}H_{23}N_2O_9PS$, N-[(R)-4-phosphopantothenoyl]-L-cysteine; $C_{17}H_{21}N_4O_9P$, Flavin mononucleotide; $C_{14}H_{18}N_5O_{11}P$, Adenylosuccinic acid; $C_7H_{15}O_{10}P$, Sedoheptulose 7-phosphate; $C_{10}H_{17}NO_3$, N-[(3s)-2-Oxotetrahydrofuran-3-Yl]hexanamide; $C_{18}H_{32}O_{16}$, Raffinose; $C_{12}H_{24}O_{11}$, Melibiitol; $C_{12}H_{22}O_{11}$, Galactinol; $C_{24}H_{42}O_{21}$, Stachyose; $C_{31}H_{45}N_6O_{17}P$, 5-Formyl-5,6,7,8-tetrahydromethanopterin; $C_{11}H_{14}N_2$, Gramine; $C_{28}H_{38}N_8O_4$, Hordatine A; $C_5H_{14}NO^+$, Choline; $C_5H_{12}O_7P_2$, Isopentenyl pyrophosphate; $C_{11}H_{14}NO_6P$, Indole-3-glycerol phosphate; $C_8H_{10}O_6$, cis-Dihomoaconitic acid,; $C_{10}H_{10}O_6$, Chorismic acid; $C_4H_{10}N_2O_2$, L-2,4-diaminobutyric acid; $C_{45}H_{62}N_6O_{12}$, Hydrogenobyrinate diamide; $C_{12}H_{24}O_{17}P_2$, bis(1L-myo-inositol) 3,1'-phosphate 1-phosphate; $C_{18}H_{23}N_5O_6$, 8-Demethyl-8-(dimethylamino)riboflavin; $C_{20}H_{22}O_6$, Matairesinol. The underline and bold entries were used to show highly significant correlation between the two data. * $p < 0.05$. ** $p < 0.01$.

Table S4. Eigenvector of eight principal components of phosphorus solubilizing ability and auxin production ability of PGPRs, plant growth status, differential metabolites in roots of tea plants, and rhizosphere microenvironment factors.

	**Principal component**							
	**1**	**2**	**3**	**4**	**5**	**6**	**7**	**8**
Phosphorus	0.825	0.507	0.166	0.020	0.046	0.138	0.104	0.053
Auxin	**0.928**	-0.165	-0.234	-0.112	0.163	0.063	0.115	0.024
Chl	0.406	-0.172	-0.029	-0.852	-0.005	0.252	0.040	-0.114
Plant height	0.739	-0.003	0.111	-0.173	0.350	-0.523	-0.124	-0.016
L-Tryptophan	**0.911**	0.049	0.062	0.092	-0.047	-0.199	-0.307	-0.141
N-[(R)-4-phosphopantothenoyl]-L-cysteine	0.700	-0.470	-0.077	-0.248	0.357	0.010	-0.286	0.112
Flavin mononucleotide	0.725	0.210	0.111	0.313	0.341	-0.100	0.253	-0.359
Adenylosuccinic acid	**0.896**	-0.170	0.171	0.029	-0.048	-0.086	-0.334	-0.128
Sedoheptulose 7-phosphate	**0.867**	0.148	0.222	0.076	-0.225	0.111	-0.112	-0.311
N-[(3s)-2-Oxotetrahydrofuran-3-Yl]hexanamide	0.717	0.491	0.142	0.164	-0.252	-0.053	0.044	0.360
Raffinose	0.710	0.534	0.229	-0.072	-0.146	0.312	-0.067	0.172
Melibiitol	**-0.924**	0.178	0.041	-0.071	0.264	0.115	0.151	0.035
Galactinol	-0.755	0.497	0.192	-0.264	0.082	0.260	0.054	-0.015
Stachyose	0.662	0.585	0.314	-0.145	0.023	0.223	0.059	0.214
5-Formyl-5,6,7,8-tetrahydromethanopterin	**-0.883**	0.322	-0.104	0.286	0.117	0.072	0.030	-0.070
Gramine	**0.906**	-0.202	-0.070	0.312	0.053	0.003	-0.167	0.075
Hordatine A	-0.703	0.033	0.624	0.144	-0.196	-0.109	0.136	0.159
Choline	0.732	0.410	0.314	0.196	-0.152	-0.127	-0.328	0.112
Isopentenyl pyrophosphate	-0.741	0.379	0.491	0.096	-0.131	0.149	0.097	-0.087
Indole-3-glycerol phosphate	-0.692	-0.578	0.257	0.244	-0.065	-0.210	0.088	0.073
cis-Dihomoaconitic acid	-0.634	0.505	0.523	0.170	-0.154	0.083	-0.045	-0.089
Chorismic acid	0.801	0.038	-0.431	0.175	0.211	-0.063	0.298	0.066
L-2,4-diaminobutyric acid	0.690	0.282	0.053	0.389	-0.003	0.131	0.466	-0.236
Hydrogenobyrinate diamide	**0.976**	-0.067	0.058	0.132	0.110	-0.057	0.074	0.032
bis(1L-myo-inositol) 3,1'-phosphate 1-phosphate	0.741	-0.566	-0.161	0.105	-0.111	0.210	0.058	0.187
8-Demethyl-8-(dimethylamino)riboflavin	0.789	0.255	0.264	0.124	0.392	0.225	-0.149	0.011
Matairesinol	**0.934**	-0.124	-0.228	-0.171	0.056	0.029	0.158	0.054
Proteobacteria	0.785	-0.076	-0.113	0.100	-0.510	-0.275	0.140	0.015
Acidobacteriota	-0.481	-0.423	0.216	-0.249	0.483	0.487	0.037	-0.098
Bacteroidota	0.357	0.489	0.149	-0.578	-0.082	-0.419	0.305	-0.027
Actinobacteriota	-0.747	0.351	-0.497	-0.097	0.048	0.145	0.190	0.046
Planctomycetota	0.670	-0.097	0.412	0.134	-0.345	0.186	-0.406	-0.190
Chloroflexi	0.459	0.180	0.193	0.706	-0.321	0.286	0.075	0.176
Verrucomicrobiota	0.130	-0.704	0.539	0.016	-0.002	0.427	-0.051	-0.106
Patescibacteria	**-0.983**	0.122	-0.047	-0.111	-0.032	0.026	-0.052	0.018
Gemmatimonadota	0.072	-0.507	0.722	-0.388	-0.086	-0.191	0.142	-0.042
Cyanobacteria	-0.050	-0.038	0.112	0.354	**0.858**	-0.230	-0.138	0.224
Zixibacteria	-0.476	0.541	-0.573	0.161	-0.067	0.172	-0.297	-0.058
Ascomycota	0.014	0.385	0.827	-0.219	0.325	-0.109	-0.008	0.045
Mortierellomycota	0.314	-0.689	-0.077	0.017	-0.616	-0.101	0.171	-0.037
Basidiomycota	-0.473	0.736	-0.041	-0.225	0.163	-0.306	-0.249	-0.016
Rozellomycota	-0.199	0.361	-0.846	0.068	-0.088	0.147	-0.254	-0.125
Chytridiomycota	-0.245	-0.667	0.106	0.475	0.440	0.161	0.044	0.194
OCC	**0.952**	-0.125	-0.167	-0.073	0.143	0.043	0.060	-0.141

TNC	**0.990**	-0.004	-0.078	-0.024	0.096	0.064	-0.001	-0.002
HNC	**0.859**	0.432	0.126	-0.012	0.104	0.173	0.113	-0.077
TPOC	0.428	-0.069	-0.053	-0.746	-0.262	0.252	-0.137	0.319
APOC	**0.920**	0.303	0.032	-0.045	0.205	-0.005	0.086	-0.102
TPHC	**0.870**	0.341	-0.219	0.013	-0.061	0.010	0.175	0.209
APHC	**0.911**	0.057	-0.164	-0.191	0.246	0.178	0.093	-0.055

Note: Determinant factors were chosen when the absolute value is greater than or equal to 0.850 and shown as underline and bold entries.
